# Current Advances and Future Perspectives of Liver-on-a-Chip Platforms Incorporating Dynamic Fluid Flow

**DOI:** 10.3390/biomimetics10070443

**Published:** 2025-07-04

**Authors:** Jingyeong Yun, Tae-Joon Jeon, Sun Min Kim

**Affiliations:** 1 Department of Biological Sciences and Bioengineering, Inha University, Incheon 22212, Republic of Korea; yunjg0618@gmail.com; 2 Department of Biological Engineering, Inha University, Incheon 22212, Republic of Korea; 3 Biohybrid Systems Research Center, Inha University, Incheon 22212, Republic of Korea; 4 Department of Mechanical Engineering, Inha University, Incheon 22212, Republic of Korea

**Keywords:** microphysiological systems, dynamic fluid flow, liver-on-a-chip, multi-organ-on-a-chip, disease modeling

## Abstract

The liver is a vital organ responsible for a broad range of metabolic functions, including glucose and lipid metabolism, detoxification, and protein synthesis. Its structural complexity, characterized by hexagonal hepatic lobules composed of diverse parenchymal and non-parenchymal cell types, supports its broad spectrum of physiological activities. Traditional in vitro liver models have contributed significantly to our understanding of hepatic biology and the development of therapies for liver-related diseases. However, static culture systems fail to replicate the dynamic in vivo microenvironment, particularly the continuous blood flow and shear stress that are critical for maintaining hepatocyte function and metabolic zonation. Recent advances in microphysiological systems (MPS) incorporating dynamic fluid flow have addressed these limitations by providing more physiologically relevant platforms for modeling liver function. These systems offer improved fidelity for applications in drug screening, toxicity testing, and disease modeling. Furthermore, the integration of liver MPS with other organ models in multi-organ-on-chip platforms has enabled the investigation of inter-organ crosstalk, enhancing the translational potential of in vitro systems. This review summarizes recent progress in the development of dynamic liver MPS, highlights their biomedical applications, and discusses future directions for creating more comprehensive and predictive in vitro models.

## 1. Introduction

The liver is a central metabolic organ responsible for maintaining systemic energy homeostasis, particularly through its regulation of glucose and lipid metabolism [1,2]. Glucose is transported into hepatocytes via membrane glucose transporters, where it is either stored as glycogen or metabolized through glycolysis into pyruvate. Pyruvate is subsequently converted to acetyl-CoA, which enters the tricarboxylic acid (TCA) cycle for ATP generation or is used in lipogenesis via citrate synthesis. In parallel, the liver synthesizes glucose via glycogenolysis and gluconeogenesis, underscoring its dual role in glucose uptake and production [1,3].

Structurally, the liver is composed of hexagonally arranged hepatic lobules, each centered around a central vein and surrounded by hepatocytes organized in radial cords. Blood from the hepatic portal vein and hepatic artery flows through fenestrated hepatic sinusoids toward the central vein, establishing oxygen and nutrient gradients across the lobule [4,5,6,7,8]. This unique architecture supports spatially zonated hepatic functions, influenced by microenvironmental cues.

The liver’s cellular composition includes both parenchymal and non-parenchymal cells. Hepatocytes (HCs), the predominant parenchymal cells, constitute approximately 60% of liver cell numbers and 80% of its volume [9]. They carry out essential functions including detoxification, bile acid synthesis, urea production, and albumin secretion—making albumin and urea key functional biomarkers [6,10,11,12]. Non-parenchymal cells such as liver sinusoidal endothelial cells (LSECs), hepatic stellate cells (HSCs), and Kupffer cells (KCs) also play critical roles in hepatic physiology. LSECs facilitate substance exchange via their fenestrations and high endocytic capacity [13,14]. HSCs, located in the space of Disse, regulate sinusoidal blood flow and contribute to fibrogenesis under pathological conditions [15]. KCs, the liver’s resident macrophages, serve as the first immunological barrier against gut-derived antigens and xenobiotics [16,17,18].

Disruptions in hepatic metabolism and detoxification pathways are implicated in a broad spectrum of liver diseases, including non-alcoholic fatty liver disease (NAFLD), alcoholic liver disease (ALD), viral hepatitis, cirrhosis, and drug-induced liver injury (DILI) [19,20,21]. Despite the increasing prevalence of these conditions, current in vitro liver models often fail to capture the complex cellular architecture, dynamic perfusion, and functional heterogeneity of the native liver microenvironment.

A variety of experimental models have been developed to investigate liver function and disease, including animal-based in vivo models and cell-based ex vivo and in vitro systems. A mouse model was used as an in vivo animal model to measure the sensitivity of metabolic capacity, including lipid accumulation in the liver due to a high-fat diet [22], and to study overall liver disease aspects, such as damage, regeneration, and repair mechanisms [23,24]. While in vivo animal models remain valuable for studying whole-organism physiology and conducting preclinical testing, they are often limited by high costs, long experimental timelines, and species-specific differences that reduce their ability to accurately predict human responses [25]. Furthermore, ethical concerns regarding animal use in research have led to increased interest in alternative approaches. In line with the 3R principles—Replacement, Reduction, and Refinement—proposed in 1959, significant efforts have been made to develop human-relevant, cell-based models that can reduce or replace animal experiments [26].

Among these advancements, the liver-on-a-chip has emerged as a promising alternative. Based on the principles of biomimetics, the liver-on-a-chip replicates the structural and functional characteristics of the human liver—such as cellular heterogeneity, tissue architecture, and dynamic fluidic environments—within a single microengineered platform. Due to its physiological relevance, this system provides a more human-appropriate experimental model, allowing researchers to investigate liver metabolism, pathophysiology, and toxicity under conditions that closely mimic the in vivo human liver environment.

This review provides a comprehensive overview of recent progress in liver in vitro modeling systems. Particular focus is given to advances in biomimetic organ-on-a-chip technologies that aim to recreate key features of the liver’s native microenvironment. Finally, current challenges and future directions in the development of physiologically relevant and scalable liver-on-chip platforms are discussed.

## 2. Cell Culture Platforms for Hepatic Cells

### 2.1. Traditional Cell Culture Platforms

Traditional in vitro liver cell culture platforms include cell culture flasks, dishes, multi-well plates, and transwell systems. Among these, cell culture flasks and dishes represent the most economical and straightforward two-dimensional (2D) culture environments. However, these systems have inherent limitations in accurately replicating the physiological conditions found in vivo. Consequently, they are primarily employed for basic cell maintenance and proliferation studies, rather than more sophisticated applications such as disease modeling or drug screening.

Well plates provide a more advanced alternative, enabling the creation of both two-dimensional (2D) and three-dimensional (3D) culture environments. These platforms are extensively utilized for high-throughput drug screening and fundamental cellular assays. Recent research has emphasized the utility of well plates in comparative analyses of cellular functions in 2D versus 3D cultures. For instance, one study employed the human liver-derived cell line (HepaRG) cultured in a 96-well plate format, treated with cadmium chloride as a model toxicant, and analyzed intracellular metabolites using nano-electrospray ionization direct infusion mass spectrometry (nESI-DIMS), thus establishing a reliable metabolomics workflow for HepaRG cells [27]. In a separate investigation, human hepatocyte-derived cell lines, human hepatocellular carcinoma cell line (HepG2) and HepaRG, were seeded in 96-well plates under static 2D conditions or cultured as 3D spheroids [28]. The cells were subsequently exposed to hepatotoxic agents, specifically amiodarone hydrochloride and acetaminophen (APAP), to evaluate DILI. This study successfully demonstrated hepatotoxicity via observed reductions in cell viability and increased liver injury biomarkers, including lactate dehydrogenase (LDH), aspartate aminotransferase (AST), and alanine aminotransferase (ALT), in response to drug treatment.

Transwells share similarities with traditional well plates but offer the distinct advantage of enabling specialized co-culture configurations, including barrier model systems. Due to their robust structural integrity, transwell platforms facilitate investigations into cell migration and mass transport across semi-permeable membranes under static conditions [29]. For example, in one study, HepG2 and a hepatocyte-derived cellular carcinoma cell line (Huh7) were cultured in the upper compartments of transwell inserts, and their migration through the underlying permeable membrane was analyzed following treatment with chronic hepatitis B medications, specifically tenofovir disoproxil fumarate and entecavir. The findings indicated that these pharmaceuticals significantly inhibited migration and invasive behavior of liver cancer cells [30]. Nonetheless, traditional well plates and transwell platforms are often inadequate for replicating the intricate structural and functional complexity of liver tissue and the dynamic intercellular interactions that occur within the hepatic microenvironment.

Due to the complexity of liver metabolism and its intricate microarchitecture, traditional in vitro models are inherently limited in their ability to accurately reproduce the physiological conditions of the in vivo hepatic environment. To overcome these limitations, organ-on-a-chip technologies have emerged, offering advanced platforms capable of closely replicating liver physiology, microenvironmental complexity, and dynamic cellular interactions.

### 2.2. Organ-on-a-Chip Platform

Organ-on-a-chip technology represents a significant advancement beyond conventional cell culture, aiming to replicate organ-level functions within a controlled in vitro environment [26]. These microengineered platforms typically include fluidic channels capable of sustaining perfusion and nutrient exchange, often lined with organ-specific cells. The structural design, materials, cell seeding strategies, and maintenance protocols of these devices can be tailored to the specific organ being modeled and the experimental objectives. In liver-on-a-chip applications, a range of human-relevant cell sources can be employed, including primary hepatocytes, liver-derived cell lines, and stem cell-derived hepatic cells [26]. This versatility allows for more accurate simulation of liver-specific architecture and functions, such as metabolic activity, bile secretion, and drug detoxification.

Overall, organ-on-a-chip systems offer a promising platform for liver modeling, with the potential to achieve higher physiological relevance and predictive accuracy than traditional static culture systems. As such, they are increasingly being adopted in drug discovery, toxicity testing, and disease modeling.

## 3. Liver-on-a-Chip Platforms Utilizing Static Culture Techniques

In in vitro experiments, the culture medium is a critical component that supports cell survival, proliferation, and function. It is typically a liquid or gel-based formulation containing essential nutrients, energy sources, and regulatory factors required for the progression of the cell cycle and maintenance of cellular activity [31]. Beyond sustaining basic cell physiology, the medium also serves as a vehicle for delivering experimental compounds, enabling controlled exposure of cells to specific stimuli according to the study design. However, as cells metabolize nutrients, they generate waste products that accumulate in the medium over time, potentially altering the local microenvironment and impacting cell behavior. Therefore, regular replacement of the medium is necessary to maintain optimal culture conditions and ensure experimental reproducibility [32].

**Figure 1:**
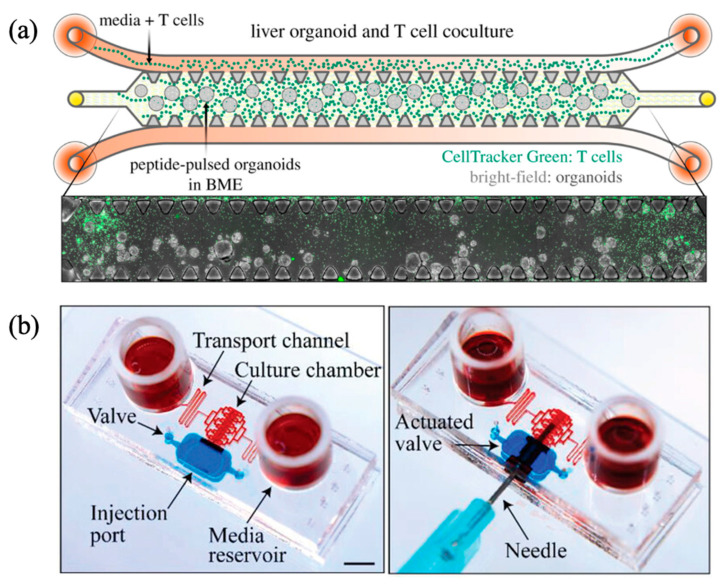
Liver-on-a-chip platform with cells cultured in a static environment. (**a**) Liver organoids and T cells were co-cultured. The T cells were mixed in the medium and injected into the medium channel. During the static incubation caused by the medium injection, the T cells mixed in the medium migrated to the middle channel in the presence of non-target peptides. Toxicity to the liver was not observed throughout the process of peptide pulsing of the T cells. Reprinted with permission from [33]. (**b**) The platform that facilitated the introduction of the liver biopsy into the culture chamber via a syringe, static culture was confirmed to be feasible, with no occurrence of medium leakage from the external environment. The injected liver biopsy demonstrated comparable functionality to freshly isolated liver tissue over a 7-day culture period, thereby suggesting the potential use as a liver-on-a-chip platform. Scale bar: 5 mm. Reprinted with permission from [34].

In certain liver-on-a-chip platforms, cell culture and media supply have been implemented under static conditions. For example, a static microfluidic chip was employed in a study investigating hepatitis C virus (HCV) infection dynamics [33]. In this system, spheroids were generated from EpCAM-positive hepatic progenitor cells isolated via liver resection, embedded within a hydrogel matrix, and injected into the central channel of a disposable 3D cell culture chip (DAX-1). In HCV research, CD8^+^ T cell is one of the key elements in controlling infection; therefore, CD8^+^ T cells were introduced into the peripheral channel. Co-culture was achieved by applying varying volumes of culture medium to the grooves flanking either side of the peripheral channel (Figure 1a). This design created a pressure gradient between the two media channels, allowing for passive diffusion that simulates blood perfusion in a static setting. Despite the absence of active flow, the system provided a spatially organized co-culture environment that enabled functional interactions between hepatic spheroids and immune cells. This approach demonstrates the potential of static liver-on-a-chip models for immunological and virological studies, particularly when mechanical simplicity and compartmentalization are required.

A polydimethylsiloxane (PDMS)-based microfluidic chip was fabricated to facilitate direct injection of liver biopsy samples via a syringe [34]. The cellular chamber was strategically situated within the central portion of the flow layer (1 mm in width, 5 mm in length, and 600 µm in height), flanked by separate valve structures on both sides. Applying negative pressure enabled the opening of these valves, permitting the insertion of a syringe needle containing liver tissue and direct deposition of biopsy material into the designated cellular compartment, thus supporting organotypic liver culture. The chip design featured tortuous microchannels on each side of the chamber, intentionally introduced to moderate fluid velocity during media exchanges. Two media reservoirs were located at the ends of these channels. Static cultures were attempted by storing 150 µL of medium in each reservoir, and the medium was refreshed every 24 h (Figure 1b). When exchanging media, the medium does not enter the culture chamber immediately but passes through the transport channel, thereby controlling the flow rate in the middle and enabling uniform delivery of nutrients. Utilizing this system, liver-specific gene expressions, including α-smooth muscle actin (α-SMA) and glial fibrillary acidic protein (GFAP), were successfully characterized over a seven-day period, and sustained hepatic functionality was demonstrated over extended culture durations. Consequently, this platform supports personalized therapeutic evaluations alongside on-chip biopsy-based assays.

A novel strategy for investigating the metabolic mechanisms of tumor cells through cell temperature measurement has recently been reported. In this approach, Ti/Pt electrodes and a silicon nitride (Si_3_N_4_) insulating layer were integrated onto a glass microfluidic chip to fabricate thermal microsensors. HepG2 cells were cultured within the C-shaped dam structure (150 μm in diameter and 36.1 μm in height) in the chip to induce hepatic metabolic activity [35]. A static culture system was employed, wherein pipette tips containing culture medium were placed at the inlet and outlet to supply nutrients and facilitate real-time metabolic measurements. Following 24 h of cell attachment, HepG2 cells displayed active metabolism and exhibited sensitivity to environmental conditions. Notably, an increase in cell temperature was observed during metabolic activity, with higher temperature elevations correlating with increased cell density.

Static culture systems have been widely utilized for cellular bioanalysis and drug testing due to their relative simplicity, ease of operation, and compatibility with high-throughput experimental designs [36]. These systems offer practical advantages for routine assays and initial screening studies. However, a key limitation of static culture is the accumulation of metabolic waste products over time, even with regular media exchange in long-term culture. This can alter the local microenvironment and negatively influence cell behavior and viability. Furthermore, the absence of perfusion and shear stress in static systems means that critical physiological stimuli—such as those generated by blood flow—are not reproduced. As a result, the metabolic activity and functional responses of liver cells under static in vitro conditions often diverge from those observed in vivo. Consequently, static culture methods fall short in replicating the complex and dynamic microenvironment characteristic of native liver tissue, limiting their utility for predictive modeling of liver physiology and disease.

## 4. Liver-on-a-Chip Platforms with Controlled Dynamic Fluidic Flow

To overcome limitations inherent to traditional static culture methods, dynamic culture approaches have been developed. These systems incorporate continuous medium flow to closely replicate in vivo hemodynamic conditions, thereby providing a more physiologically accurate tissue microenvironment. The introduction of dynamic flow significantly enhances the distribution and diffusion of nutrients and oxygen throughout the culture, while also facilitating efficient removal of cellular metabolic waste. These enhancements result in increased cell viability, improved metabolic activity [37], and enhanced cellular migration [38] compared with static culture conditions.

The liver is particularly sensitive to microenvironmental factors, exhibiting spatial gradients of oxygen, nutrients, and signaling molecules that regulate its metabolic zonation and functional heterogeneity [39]. Static culture platforms inherently fail to replicate these gradients or the associated shear stresses, limiting their ability to accurately model liver-specific functions and cellular responses. Conversely, dynamic culture systems better preserve hepatic functionality, including increased albumin secretion, enhanced urea production, and improved cytochrome P450 enzyme activity [40,41].

Consequently, dynamic culture methodologies are increasingly adopted in liver-related in vitro studies to achieve greater biological relevance. Liver-on-a-chip platforms utilizing perfusion-based dynamic systems effectively recreate flow conditions, allowing hepatocytes to respond more accurately to biochemical and mechanical stimuli, thus enhancing the fidelity of functional assays. In the organ-on-a-chip field, dynamic flow is commonly implemented through auxiliary mechanisms such as pumps, tilting platforms, or gravity-driven fluid delivery systems. Recent liver-on-a-chip studies employing various dynamic flow strategies are summarized in Table 1, with select examples discussed in greater detail below.

### 4.1. Dynamic Fluid Flow Driven by Gravity Gradient

Media perfusion can be achieved by setting a gravity gradient on liver-on-a-chip platforms. In this case, external equipment can be eliminated by maintaining a consistent height difference between the media reservoirs. Inspired by the physiological structure of hepatic lobules, a liver chip was designed to mimic blood flow patterns present in both portal and central veins in vivo [43]. In this approach, differentiated human hepatic progenitor HepaRG cells, along with human hepatic stellate cells (LX2) and human hepatic sinusoidal endothelial cells, were encapsulated within a scaffold-free three-dimensional extracellular matrix prepared from a fibrinogen solution combined with thrombin. This cellular mixture was subsequently injected into the lower culture compartment of a PDMS-based chip.

Media flow through the chip was maintained by injecting culture medium into channels representing the portal vein, hepatic artery, and central vein via pipette tips inserted into reservoirs positioned at varying heights, thus creating controlled gravity-driven flow dynamics within the culture chamber. The medium was replenished daily to sustain nutrient delivery and waste removal. Beginning on day 4 of culture, introduction of adipogenic media enabled the platform to recapitulate lipid zonation characteristic of liver lobules and accurately model the progression of NAFLD. Due to its structural fidelity—particularly the incorporation of dual blood supply features—the chip closely mimics physiological liver architecture and function, thereby representing a highly relevant in vitro model for studying NAFLD and related hepatic disorders.

Microfluidic devices have been engineered to effectively replicate the structural and functional attributes of the liver. Specifically, a PDMS-based liver chip has been developed, incorporating a periportal region that simulates hepatic triads and a perivenous region mimicking the central vein [42]. The device includes a partially radial core structure that closely resembles the three-dimensional architecture of the liver acinus. HUVECs and HepG2 hepatocytes were seeded onto this central structure (80 μm in width at the entrance, 90 μm in width at the end, and 67 μm in height), supported by medium reservoirs represented by peripotal (PP) and perivenous (PV) wells situated on either side. By maintaining a higher fluid level in the PP reservoirs (300 μL) compared to the PV reservoirs (150 μL), effective compartmentalization of nutrients and metabolites was achieved. Optimized medium delivery was critical for sufficient oxygen and nutrient supply to cells cultured within the chip. This platform contributes significantly to liver research by enabling experimental demonstration of hepatic zonation through a straightforward fabrication process, as well as by simulating structural necrosis induced by hepatotoxic agents.

**Figure 2:**
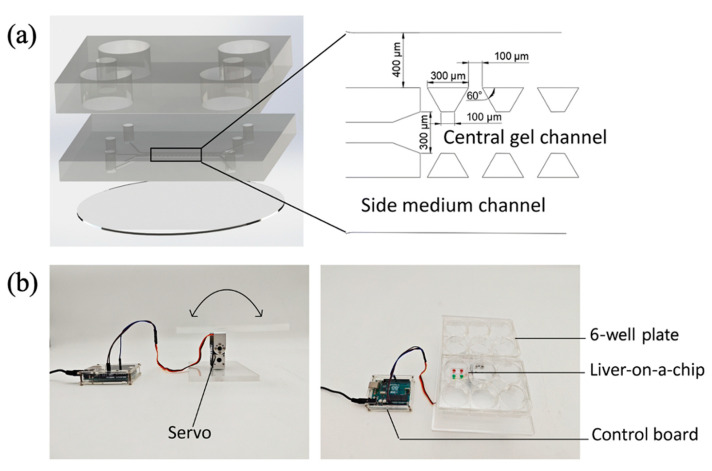
(**a**) Design schematic of the liver-on-a-chip platform. The chip comprises three layers: a top medium reservoir, an intermediate cell culture layer, and a glass bottom substrate. The cell culture layer is spatially compartmentalized into a central gel-containing channel flanked by medium channels on each side. (**b**) A rocker mechanism induces media perfusion through gravity-driven bidirectional flow. Reprinted with permission from [44].

However, dynamic flow within liver-on-a-chip systems is typically generated using external devices such as rockers or rotators that leverage gravity gradients to mimic physiological blood circulation. For example, a rocker-based liver-on-a-chip was designed to investigate DILI [44]. In this setup, HepG2 cells were encapsulated within a collagen matrix and injected into the central channel of a PDMS chip featuring a three-channel layout. A suspension of LX2 was introduced into one side channel, while the other side channel was perfused with cell-free medium, thereby establishing a co-culture environment. During incubation, medium reservoirs were filled, and the chip was positioned on a rocker inside an incubator, providing intermittent, bidirectional flow by cyclically tilting the chip at a 7° angle every seven minutes (Figure 2). Equilibrium between media reservoirs due to height differences was achieved within approximately one minute after tilting. The dynamic, oscillatory flow conditions within the chip supported robust cellular function, confirmed by assays for CYP3A4, albumin secretion, and vimentin expression. Moreover, hepatotoxic responses induced by APAP, aristolochic acid I (AA), and aristolactam AII (AL) were evaluated, revealing substantial differences in cellular responses between the liver-on-a-chip and conventional 2D cultures. This comparative analysis highlighted the practical advantages of the rocker-based liver chip, demonstrating its simplicity, effectiveness in promoting cellular viability and functionality, and utility in reliable drug screening applications.

In a related study, a rocker-based liver-on-a-chip system was utilized to co-culture primary HCs, HSCs, LSECs, and KCs [46]. HCs, HSCs, and KCs were encapsulated within collagen gel and injected into the central channel of a triple-channel microfluidic device, fabricated from PDMS and cover glass substrates. LSECs were subsequently seeded onto surfaces facing the adjacent side channels. To model hepatic steatosis, hepatocytes were exposed to oleic and palmitic acids, inducing free fatty acid (FFA) overload and subsequently NASH. Further inflammatory conditions were simulated by the introduction of lipopolysaccharide (LPS). During incubation, the microfluidic chips were placed on a rocker to achieve gravity-driven media perfusion, with medium replacements conducted every two days to maintain consistent head pressure. Exposure to the combination of FFAs and LPS resulted in increased numbers of cleaved caspase-3-positive cells and enhanced lactate dehydrogenase (LDH) activity. Notably, the NASH-on-a-chip model sustained disease-specific conditions over a 14-day period, characterized by elevated fibrotic protein expression in HSCs and progressive elevation of inflammatory biomarkers. The rocker mechanism facilitated straightforward collection of culture medium samples for the analysis of NASH markers, emphasizing the platform’s utility for disease modeling and drug screening applications.

In another application, the commercially available OrganoPlate 2-lane chip (MIMETAS, Leiden, Netherlands) was adapted as a high-throughput hepatotoxicity assessment platform, utilizing a rocker to control medium perfusion [47]. The liver microfluidic device incorporated induced pluripotent stem cell (iPSC)-derived hepatocytes (iCell 2.0 hepatocytes), KCs, THP-1 monocytes, and human microvascular endothelial cells (HMEC-1). The iPSC-derived hepatocytes were embedded in collagen gel and introduced into dedicated cell culture channels, while HMEC-1 and THP-1 cells were seeded at an angle, lining the adjacent perfusion channels to form a multicellular liver model. Media reservoirs in the perfusion channels were replenished periodically, with controlled gravity-driven flow induced by tilting the device up to 15° every four minutes within a perfusion rocker platform. Hepatocytes in this setup maintained high viability for up to 15 days and exhibited robust albumin secretion. Exposure to hepatotoxins troglitazone and aflatoxin B1 for three days demonstrated dose- and time-dependent hepatotoxic responses. Furthermore, the evaluation of drug metabolism confirmed the system’s potential as an effective high-throughput drug screening model, compatible with standard laboratory equipment.

**Figure 3:**
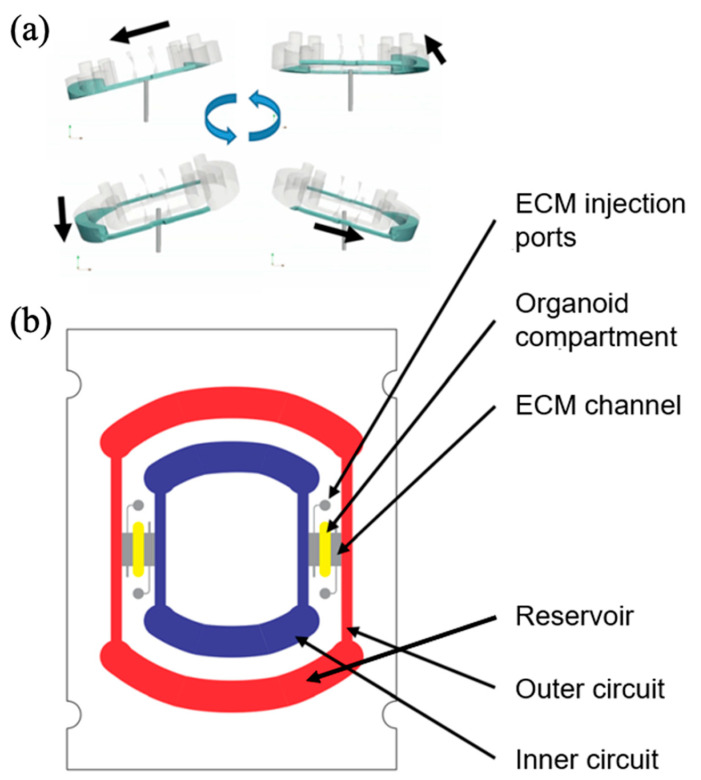
Overview of a pump-less organ-on-a-chip. (**a**) A circular-like structure is set up to guarantee that the fluid inside the platform moves unidirectionally without backflow and drying based on 3D tilting. A rotator is introduced as an external device. (**b**) Detailed structure of the chip. When stem cell-derived liver organoids were cultured in the organoid compartment, they exhibited physiologically relevant levels of albumin and urea secretion and drug metabolism. Reproduced from [50] with permission from the Royal Society of Chemistry.

However, rocker-based platforms typically generate short, bidirectional flows that may not fully replicate physiologically relevant unidirectional blood flow [50]. To address this limitation, an alternative approach utilized a standard laboratory rotator device in a liver-on-a-chip platform to achieve continuous, unidirectional medium flow (Figure 3a). The device featured an elongated and vertically positioned media reservoir, intentionally designed to generate stable, unidirectional flow. Additionally, a ring-shaped perfusion channel enabled effective circulation of culture media within the chip (Figure 3b). Flow characterization experiments verified unidirectional fluid motion without any bidirectional or reversing flow patterns. Hepatic organoids, differentiated from human pluripotent stem cells, were injected into the organoid compartment (0.5–1.2 mm in width, 4 mm in length, and 0.8/0.9 mm in height) and incubated on the rotator device for up to two weeks. Long-term maintenance of hepatic function was confirmed by sustained secretion of albumin and urea. This simplified system allowed cell loading and media replenishment via pipetting, and its design effectively trapped air bubbles at the top of the reservoir, minimizing their impact on fluid flow. The absence of external control devices such as pumps enhanced the economic viability, ease-of-use, and scalability of the platform, highlighting its suitability for routine laboratory use.

### 4.2. Unidirectional Medium Perfusion by Pump Systems

Among dynamic flow methodologies employed in liver-on-a-chip systems, pumps represent the most commonly preferred approach. Unlike rockers and rotators discussed previously, pumps provide superior precision in controlling flow rates and patterns. The continuous, regulated supply of culture medium ensured by pumps maintains a stable experimental environment and permits a broad range of customizable flow profiles.

The selection of an appropriate pumping mechanism is crucial for establishing physiologically relevant cell and tissue cultures. Pumps employed in liver-on-a-chip systems generally fall into three categories: syringe pumps, peristaltic pumps, and pneumatic pumps. Each of these pump types enables continuous, unidirectional perfusion or circulation of cell culture medium, thereby supporting sustained cell viability and functional maintenance [66]. Recent studies have successfully demonstrated the efficacy of these pump-driven platforms in liver-on-a-chip applications.

**Figure 4:**
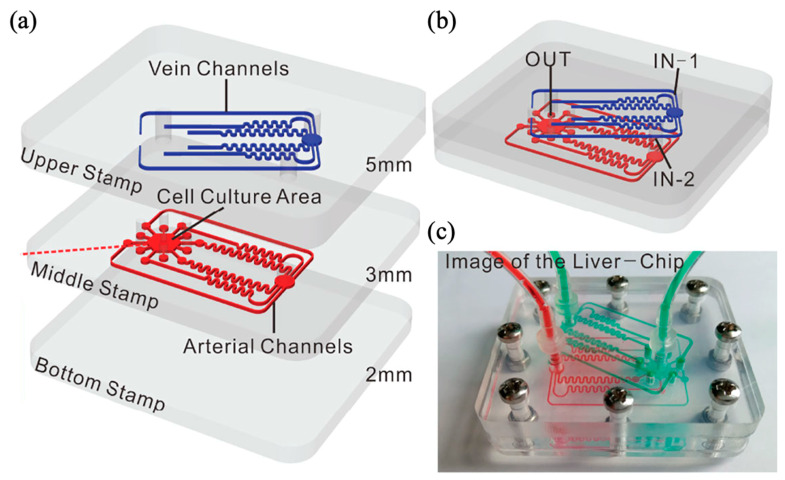
PMMA liver-on-a-chip constructed by focusing on the triad of vessels in the liver. (**a**) A total of 6 microchannels for medium perfusion are inserted into the top layer and middle layer, which have hexagonal cell culture zones. Each channel has the same flow resistance. (**b**) View of the structurally completed chip and (**c**) image of the chip under perfusion. Reprinted from [51] with open access.

A representative example is a dynamic liver-on-a-chip platform designed to replicate the hepatic vascular network [51]. This device was fabricated from polymethyl methacrylate (PMMA) and comprised three layers with embedded microchannels (Figure 4). The PMMA-based chip modeled three key hepatic vessels: the hepatic artery, hepatic portal vein, and central vein. Each channel in the top and middle layers was designed with identical fluidic resistance, converging into a central hexagonal cell-culture compartment. LSECs mixed with collagen, hepatocytes, and microspheres were injected into the compartment, forming a perfusable vascularized tissue model. Medium perfusion at a flow rate of 200 μL/h was implemented using a syringe pump, providing consistent nutrient and oxygen delivery. Computational fluid dynamics (CFD) simulations using COMSOL software confirmed physiologically relevant concentration gradients of oxygen and glucose, validating the suitability of the chip for dynamic vascular modeling. This design holds significant promise for investigating phenomena such as tumor metastasis within an accurately replicated hepatic microenvironment.

In another study, a microfluidic device was engineered to mimic liver microarchitecture, integrating cell culture chambers surrounded by interconnected media channels separated by an array of 2 μm wide slits to immobilize HepaRG hepatocyte-like cells [52]. The slit design effectively retained cells within the culture chamber while enabling controlled media perfusion. Based on previous findings indicating that hepatocytes cannot tolerate shear stresses exceeding 0.5 Pa [67] and considering oxygen diffusion through PDMS, culture medium was perfused through the device’s media channel at a controlled flow rate of 375 nl/min using a syringe pump connected to a media outlet tube. Under these conditions, HepaRG cells maintained their viability for at least 14 days, demonstrating the suitability of this approach for long-term hepatic cell culture. Importantly, spontaneous differentiation of HepaRG cells within this precisely engineered environment was observed, demonstrating successful creation of a biologically relevant hepatic structure under optimized fluidic conditions.

**Figure 5:**
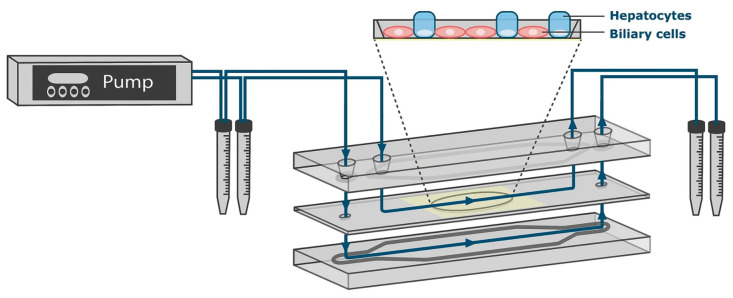
Design of a microfluidic device incorporating a pneumatic pump. Culture medium is perfused through the central and lower channels of a three-layered microfluidic platform, supporting differentiation of HepaRG cells into hepatocyte- and bile duct epithelial-like cells. Medium flow is driven by air pressurization via a pneumatic pump. Reprinted with permission from [53].

A further investigation utilized an air-pressurized pneumatic pump to achieve medium perfusion in a multilayered glass microfluidic device [53]. HepaRG cells cultured on a porous membrane located beneath a central flow channel experienced continuous medium flow maintained at a constant rate of 100 μL/h within a closed-loop system (Figure 5). Comparative analyses were conducted against static conditions in conventional well-plate cultures and primary hepatocytes. Evaluations of gene expression, cell density, and CYP450 enzyme activity over 4- and 8-week periods, along with responses to hepatotoxic compounds (ethoxyresorufin, TCDD, and rifampicin), confirmed that the dynamic microfluidic culture more closely resembled primary hepatocyte physiology than static controls. The pump-based perfusion significantly enhanced the physiological relevance of the model, establishing its utility for chronic hepatotoxicity assessments and highlighting the feasibility of applying dynamic flow conditions in long-term drug toxicity studies.

### 4.3. Medium Circulation Mediated by Pump Systems

Given the continuous circulation of blood in the human body, medium circulation within liver-on-a-chip platforms represents a promising strategy for replicating physiological conditions more accurately. Indeed, multiple studies have demonstrated the utility of medium circulation systems in liver MPS.

**Figure 6:**
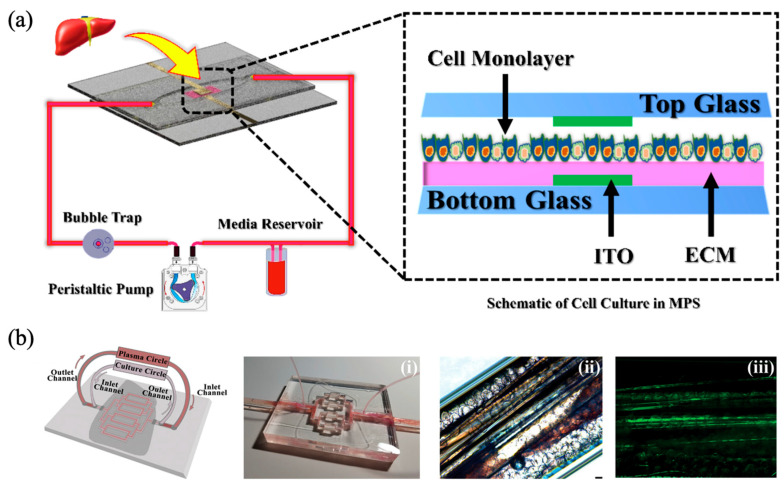
Design of a liver MPS containing a TEER sensor. (**a**) The media reservoir, peristaltic pump, bubble trap, and biochip are connected by tubing to allow continuous circulation of the media. Hepatocytes were attached to the inner bottom surface in a monolayer and included an ITO TEER sensor to evaluate their density, tissue growth, and distribution. Reprinted from [59] under open access. (**b**) Design and (**i**) image of a BBALS. The culture medium circulation circuit and plasma filtration circuit are in one system. (**ii**,**iii**) Images of the effective implementation of functional units of BBALS. Scale bar: 100 μm. Reprinted with permission from [62].

In a comparative analysis, various ECM components, including rat-tail collagen type I, fibronectin, poly-L-lysine, and Matrigel, were evaluated under varying concentrations to determine their effectiveness in supporting hepatic tissue development and tight junction protein (TJP) expression in a liver MPS [59]. HepG2-loaded MPS constructs were fabricated on glass chips equipped with indium tin oxide (ITO) sensors for transepithelial electrical resistance (TEER), placed at the top and bottom of the cell compartments. These MPS units were integrated with an external peristaltic pump and tubing, forming a closed-loop medium circulation system (Figure 6a). The system operated at a flow rate of 60 μL/min in the microfluidic channel (800 μm in width, 300 μm in height), maintaining a shear stress of 0.5 dyne/cm^2^. Hepatocyte functionality was assessed by measuring albumin secretion, urea production, and CYP450 enzyme activity. ECM effects were further characterized by imaging to quantify cell attachment and retention, and TEER measurements were used to evaluate cell density and integrity. Among the tested ECMs, Matrigel provided the highest TEER values, indicative of enhanced hepatocyte adhesion and integrity.

To assess the scalability and feasibility of fabricating liver-on-a-chip systems, biochips were evaluated for their performance in dynamic culture conditions [57]. The chips were manufactured using two methods: replica molding of PDMS and injection molding of COC. HepG2/C3A cells were seeded onto the inner surfaces (100 μm in height) of the biochips and allowed to adhere for 24 h. The chips were then integrated into a perfusion system equipped with a peristaltic pump and a bubble trap, and culture medium was continuously perfused at a flow rate of 25 µL/min. To assess flow dynamics, a range of flow rates and pressures was tested, and cells were cultured for two durations—3 and 8 days—to represent short-term and long-term conditions. Throughout the culture periods, key parameters such as cell adhesion, viability, and albumin production were monitored. Cell viability exceeded 90% in both short- and long-term cultures, and cytoskeletal integrity was confirmed through phalloidin staining of actin filaments. Furthermore, the absorption characteristics of the chip materials with respect to drugs and chemicals were evaluated, confirming the suitability of the biochip as a reliable platform for drug testing applications.

Recognizing the importance of three-dimensional (3D) spheroid formation in liver-on-a-chip systems, hydroscaffolds containing hyaluronic acid, RGDS peptides, galactosamine, and collagen were integrated into chip platforms to emulate in vivo hepatic extracellular matrix conditions [63]. Medium circulation was facilitated through a closed-loop system using a peristaltic pump at a flow rate of 10 µL/min for 20 days. By day 21, extensive spheroid formation by HepG2/C3A cells was observed. Immunostaining confirmed expression of bile salt export pump (BSEP) and multidrug resistance-associated protein 2 (MRP2), indicative of bile canaliculi formation. Additionally, hepatocyte functionality, including albumin secretion and urea synthesis, was validated. Collectively, this study underscored the feasibility of employing peristaltic pump-driven medium circulation for medium-throughput Integrated Dynamic Cell Culture Microsystem assays.

To model acute liver failure (ALF), a condition characterized by sudden hepatic dysfunction and extensive hepatocyte necrosis, a biomimetic bioartificial liver system (BBALS) was developed using hepatocytes derived from hiPSCs. Gelatin methacryloyl (GelMA) microparticles served as cell carriers, facilitating hepatocyte attachment and subsequent spheroid formation. These spheroids were integrated with ethylene-vinyl alcohol (EVOH) semi-permeable microtubes, forming the foundational structure of a 3D PDMS-based liver-on-a-chip. The device incorporated two distinct circulation circuits, each driven by peristaltic pumps: one for plasma and another for culture medium (Figure 6b). Hepatocyte viability was sustained within this system, and cellular functionality was validated through ammonia detoxification, oxygen consumption, and glucose metabolism assays. Experimental results obtained from plasma filtration studies using an ALF rabbit model highlighted the potential of this BBALS, establishing a valuable foundation for future developments in ALF therapeutics [62].

In another investigation, continuous medium perfusion via a peristaltic pump was employed to model hepatic disease progression [61]. In this approach, HepG2 hepatocytes were cultured in withdrawable wells containing porous nylon membranes at their base. HUVECs were cultured on microfluidic channels beneath these membranes, effectively replicating liver compartments and associated vascular structures. This arrangement was validated as physiologically relevant through evaluations of cell viability, barrier integrity, protein secretion, and reactive oxygen species (ROS) generation. Additionally, hepatocyte-containing wells were perfused with media supplemented with varying concentrations of FFAs to simulate the progression of NASH within the chip’s liver compartments. Significantly, intravenous drug administration was simulated by circulating drug-containing media through the perfusion system.

Peristaltic pump-based systems have gained popularity due to their inherent advantage of isolating fluids from direct contact with the pump components, thereby minimizing contamination risk. Although maintenance is relatively simple, these systems require intricate equipment configurations due to external tubing and may introduce pulsatile flow, potentially impacting cellular behavior. Therefore, alternative pumping mechanisms have been explored to control medium flow in liver-on-a-chip systems.

**Figure 7:**
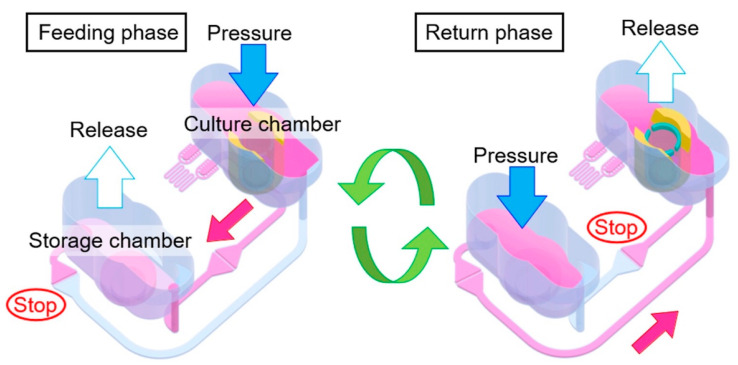
Medium circulation mechanism in a pressure-driven MPS. The cyclic repetition of feeding and return phases enables unidirectional medium flow (red arrows) through channels regulated by Laplace valves. This preserved multilayered liver tissue within the device, demonstrating potential in maintaining hepatic functionality at high cell density. Reprinted with permission from [65].

One such alternative involved developing a pneumatically driven MPS (Figure 7). This device comprised two primary compartments: a cell culture chamber and a media storage compartment, interconnected through pneumatically controlled channels. Medium circulation was facilitated by adjustable internal pressure, which regulated media levels between compartments. Flow between the compartments was directed through supply/bypass and return channels, each equipped with Laplace valves designed to open only upon achieving specific pressure thresholds, thus preventing unintended media flow. HepG2 hepatocytes were cultured at densities approximately 70-fold greater than conventional culture dishes, resulting in multilayered cellular structures. Following cell attachment, medium perfusion at rates of approximately 12–14 μL/min was achieved. Over a 20-day culture period, hepatocytes maintained metabolic activity with albumin secretion and urea synthesis slightly lower than static controls, but high relative to the device’s limited surface area. This demonstrated the efficacy and physiological relevance of the pneumatically regulated liver-on-a-chip platform [65].

## 5. Multi-Organ-on-a-Chip Systems Incorporating a Liver Compartment

Although liver-on-a-chip models effectively replicate the dynamic flow characteristics of blood circulation through perfusion and continuous medium exchange, they have inherent limitations. In vivo, the liver occupies a central role in metabolic processes; however, its functions are profoundly influenced by interactions with other organs. To more accurately capture these complex inter-organ relationships, multi-organ-on-a-chip platforms have emerged as powerful tools. These advanced platforms integrate multiple organ models, such as the liver, intestine, pancreas, lungs, and kidneys, within a unified microfluidic system. Media perfusion circuits connecting these distinct organ compartments enable simulation of inter-organ communication and metabolic interactions. Consequently, extensive studies have been conducted comparing monoculture versus co-culture systems within organ-on-a-chip platforms to provide more accurate disease modeling and drug-testing outcomes. Table 2 provides a concise summary of representative studies that have successfully co-cultured liver cells with cells from other organs.

For investigating insulin dynamics and glucose responses linked to type 2 diabetes and NAFLD, a liver–pancreas-on-a-chip was developed by integrating these two key organs into a unified perfusion-based microfluidic device [69]. This device was fabricated from PMMA, PDMS, and off-stoichiometric thiol-ene-epoxy (OSTE+). Operated pneumatically by a pump, the device comprised a central media compartment and two satellite compartments housing primary hepatocytes and pancreatic islet spheroids, respectively. Each zone is designed to hold 40 µL of medium in equilibrium. Physics-based computational modeling indicated optimal shear flow rates close to physiological conditions, suggesting flow rates of 100 µL/min for hepatocytes and 50 µL/min for pancreatic cells, achieved through specifically configured channel geometries. Insulin secretion dynamics and glucose-responsive gene expression were assessed by exposing the integrated device to low and high glucose concentrations. High glucose exposure led to downregulation of insulin-responsive genes in liver spheroids, highlighting the capability of the device to model physiological glucose–insulin interactions accurately without external insulin supplementation.

A liver-intestine-on-a-chip study introduced a pneumatically driven circulation system, pioneering co-culture experiments involving hiPS-derived intestinal cells and fresh human hepatocytes [73]. The two compartments to culture intestinal cells and human hepatocytes isolated from PXB-mouse were interconnected by microfluidic flow channels (1000 μm in width and 298 μm in depth), with pneumatic pressure alternately applied to each compartment, ensuring unidirectional flow via a narrow Laplace valve (110 μm in width and 27 μm in depth). Metabolic activity was evaluated by liquid chromatography–tandem mass spectrometry (LC-MS/MS), revealing enhanced cytochrome P450 enzyme activity in hepatocytes within the co-culture compared to monoculture conditions. Intestinal cells maintained high TEER values and displayed marker expression comparable to monocultures, confirming effective inter-organ interactions and potential for broader applications.

**Figure 8:**
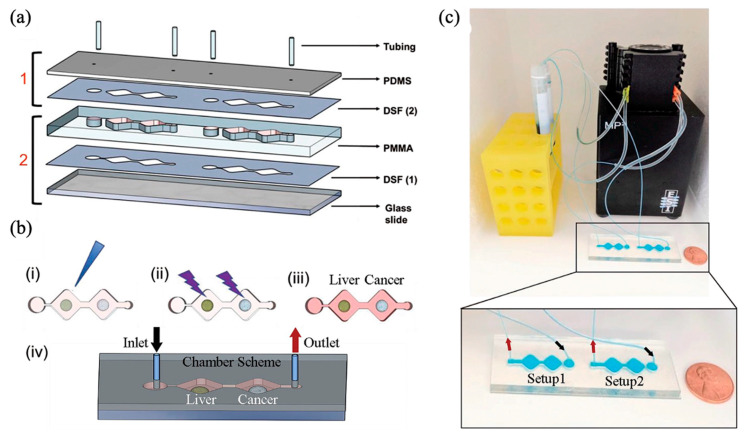
Schematic design of a chip system containing two organs. (**a**) The chip is divided into a (1) top and (2) bottom, with media flowing in and out through tubing. (**b**) Representing the stages of generation of the functionally active liver and tumor spheroids. (**c**) Experimental setup. A total of two systems on one chip, with media in the reservoir circulating due to a peristaltic pump. Reprinted with permission from [72].

Another liver-intestine-on-a-chip was fabricated using PDMS, double-sided adhesive film (DSF), and PMMA (Figure 8a), integrating a liver compartment and a cancer compartment [72]. The liver compartment housed organoids composed of human-induced hepatocyte-like cells, primary human KCs, and LSECs, while the cancer compartment contained spheroids formed from the human colon cancer cell line HCT-116 (Figure 8b). Culture medium circulated continuously at 4 µL/min via a peristaltic pump-driven closed-loop system (Figure 8c). Human primary hepatocytes served as positive controls, while fibroblasts acted as negative controls to validate hepatic metabolic functionality. Metabolic activity was demonstrated through the conversion of the anticancer prodrug capecitabine within the liver compartment, following exposure for 6 days. These findings underline the practical applicability of this liver–intestine-on-a-chip model for drug discovery purposes.

For simultaneous modeling of liver and kidney interactions, a single integrated microfluidic platform was developed [75]. The aim was to examine whether small EVs derived from mesenchymal stromal cells (known for their renoprotective effects) could potentially induce off-target effects in the liver. Utilizing the commercial microfluidic device, human biopsy-derived liver organoids and renal tubuloids were cultured in separate compartments connected within a unified microfluidic circuit. Fluid flow was maintained for eight days through peristaltic conditions created by three pneumatic pumps operating alternately. The cell compartments were designed with open tops to facilitate media exchange every 2–3 days. Results demonstrated EV accumulation in both kidney and liver compartments, with liver accumulation notably influenced by kidney injury status. This platform thus effectively demonstrated inter-organ interactions and provided a valuable tool for therapeutic EV development.

## 6. Conclusions and Future Perspectives

The liver functions as a central metabolic hub, consisting of hepatic lobules and various specialized cells involved in critical processes such as glucose and lipid metabolism. Disturbances in these metabolic pathways can result in hepatic injury and diseases, including NAFLD, ALD, hepatitis, cirrhosis, and DILI. Due to the global health impact of these conditions, there is an ongoing necessity for detailed investigation. Traditionally, animal models have been extensively used to study liver pathophysiology. However, ethical considerations, cost, and limitations in translating results to humans have led researchers to shift toward in vitro cell-based models.

Emerging from traditional 2D monolayer cultures or advancing into 3D culture systems, liver-on-a-chip technology offers significant enhancements over conventional culture platforms like well plates and transwells. A notable advancement in this technology is the integration of controlled medium flow. Two primary flow mechanisms have been developed for microfluidic systems: gravity-driven and pump-driven approaches. Gravity-driven systems are simple and cost-effective, but offer limited control over flow dynamics. In contrast, pump-driven systems enable precise regulation of flow rates, ensuring a consistent supply of culture media. Early pump-driven systems typically implemented unidirectional flow; however, to better replicate physiological blood circulation, many platforms have since adopted continuous recirculating flow systems using pumps. These advancements have facilitated the development of sophisticated liver-on-a-chip models incorporating various cellular configurations, including 3D cell cultures, spheroids, and organoids. Such models offer enhanced capabilities for accurate disease modeling and predictive drug testing.

Functionally, the liver extensively interacts with other organs in the body, prompting the development of multi-organ-on-a-chip systems. These platforms incorporate various organs such as the intestine, pancreas, lungs, kidneys, and skin alongside the liver. Research has demonstrated that co-culture of the liver with other organs can provide more physiologically relevant and reliable outcomes compared to single-organ models, especially in drug screening applications.

This review has primarily focused on recent advancements in dual-organ systems, particularly those involving liver integration. However, ongoing research efforts are expanding to include multi-organ-on-a-chip technologies connecting three or more organs. As these platforms continue to evolve, the inclusion of additional organs beyond the liver will further enhance their predictive accuracy. Moreover, systematizing cell culture approaches will contribute to developing standard guidelines, thus improving reproducibility and comparability across studies. Additionally, artificial intelligence (AI) and machine learning (ML) technologies can be introduced to increase experimental efficiency. Integrating organ-on-a-chip technology with AI and ML for experimental protocol validation or data analysis will contribute to the transformation of laboratory-based innovations into high-efficiency, industrial-scale applications, thereby significantly advancing drug screening methodologies.

## Figures and Tables

**Table 1:** Liver-on-a-chip platform with dynamic flow.

Flow Control Strategy		Cell Type		Description	Ref.
Gravity gradient (Pump-less)	Height difference between medium reservoirs	HepG2, human umbilical vein endothelial cell (HUVEC)	3D	A volume difference of 150 μL was given between the two reservoirs	[42]
				Reproducing hepatic zonation, assessing APAP toxicity	
		HepaRG, LX2, human hepatic sinusoidal endothelial cell (HHSEC)	3D	A height difference of 15–18 mm was given between storage containers	[43]
				Modeling NAFLD, reproducing dual hepatic lobular perfusion	
	Rocker	HepG2, LX2	3D	Tilting ± 7°, every 7 min	[44]
				Assessing the effects of aristolochic acid I (AA) and aristololactam AII (AL)	
		Primary human hepatocytes (PHH), HUVEC	3D	Tilting ± 15°, 1 rpm velocity	[45]
				Recapitulating vascularized liver structure	
		Primary human HC, HSC, KC, LSEC	3D	Modeling the progression of non-alcoholic steatohepatitis (NASH), assessing the toxicity of elafibranor	[46]
		iCell 2.0 Hepatocytes, THP-1, HMEC-1	3D	Tilting to a maximum angle of 15°, every 4 min	[47]
				Metabolic analysis of phenacetin, coumarin, diclofenac, terfenadine, phenolphthalein	
		Upcyte^®^ Human Hepatocytes, HUVEC	3D	Tilting ± 14°, every 8 min	[48]
				Simulating veno-occlusive disease	
		iCell^®^ Hepatocytes 2.0, primary liver-derived endothelial cell (LDEC), primary HSC, HUVEC	3D	Tilting ± 14°, every 8 min	[49]
				Metabolic dysfunction-associated steatohepatitis (MASH) modeling, Analysis of the effects of SB-431542 and firsocostat	
	Rotator	Human embryonic stem cell, human-induced pluripotent stem cell (hiPSC), HUVEC	3D	Tilting 10–17°, 1–5 rpm speed	[50]
				Suggesting the possibility of circulating immune cells in the device	
Perfusion	Syringe pump	Murine primary HCs, LSECs LX2	3D	2 mL/h flow rate in channel containing cells	[51]
				Designed triple vessels in the liver and analyzed the effects of glucose concentration gradients	
		HepaRG	3D	375 nl/min flow rate	[52]
				Reproduction of the liver microstructure	
	Air-pressurized pump	HepaRG, PHH	2D	100 µL/h flow rate	[53]
				Demonstration of enhanced CYP metabolic activity of hepatocytes, characterization of HepaRG cells	
	Microfluidic pump	HepG2	3D	The inlet velocity is optimized to 10^−5^ m/s	[54]
				Replicating hepatic lobule structure	
	Peristaltic Pump	HepG2, HUVEC	2D & 3D	99 μL/min flow rate	[55]
				Reproducing acute liver failure, assessing APAP toxicity	
Circulation	Peristaltic flow	HepG2, CCD-986sk	2D	50 μL/min flow rate	[56]
				Integrated an electrochemical albumin measuring sensor and modeled diabetes to evaluate metformin	
		HepG2/C3A	2D	25 µL/min flow rate	[57]
				Evaluating dynamic cell culture on cyclic olefin copolymer (COC) biochips	
		Primary rat hepatocytes, HSCs, LSECs, KCs	2D	1.5 mL/min flow rate	[58]
				Modeled advanced chronic liver disease (ACLD), inducing dysregulation of hepatic endothelium-related pathways	
		HepG2	2D	60 μL/min flow rate	[59]
				Assessing extracellular matrix (ECM) suitable for cell adhesion and the microenvironment reproduction	
		PHH, HUVEC	2D	14 µL/min flow rate	[60]
				Reproduced the structure, microenvironment, and function of the liver	
		HepG2, HUVEC	2D	300 μL/min flow rate	[61]
				Modeling NASH progression, evaluating antihyperlipidemic drugs	
		hiPSC-Heps, PHH	3D	0.5, 1, and 2 mL/min flow rate	[62]
				Developed a bioartificial liver (BAL) system with validated plasma filtration capabilities	
		HepG2/C3A	3D	10 µL/min flow rate	[63]
				Confirmed the functionality of 3D liver chips on hydroscaffolded biochips	
		H3CX	3D	Fabrication of microvascular networks	[64]
	Pneumatic flow	HepG2	3D	12–14, 3.4 μL/min flow rate in feed channel and bypass channel, 350–800 μL/min flow rate in return channel	[65]
				Evaluation of the functionality of HepG2 in a 3D stacked system	

**Table 2:** Multi-organ-on-a-chip platforms with liver with medium circulation system.

Flow Control Strategy		Cell Type		Description	Ref.
Circulation	Peristaltic flow	hiPSC (liver and islet)	3D	100 µL/h flow rate	[68]
				Evaluating the effects of high glucose on glucose–insulin-associated function and the effect of metformin	
	Pneumatic Flow	primary human cell (liver and islet)	3D	Various flow rated from 0 to 4000 µL/min	[69]
				Mimicking high glucose environment, observing changes in insulin sensitivity and glucose responsive genes	
Gut (Intestine)	Rocker	HepG2 (liver), Caco-2 (intestine)	2D	Tilting ± 10° every 5 min, 240 μL/h and 80 μL/h flow rates in gut and liver channels	[70]
				Modeling hepatic steatosis, assessing the impact of gut-liver interactions on the anti-steatotic effects of XL-335 and metformin	
	Peristaltic flow	HepG2 (liver), Caco-2 (intestine)	2D	Flow rate controlled within 0 to 20 nL/min using pump on/off frequency	[71]
				Modeling NAFLD using FFA	
		human induced hepatocyte-like cells, HLKC, HLECP2 (liver), HCT-116 (colon cancer)	3D	4 μL/min flow rate	[72]
				Evaluating the decreased survival of tumor when capecitabine is metabolized in the liver	
	Pneumatic flow	Human hepatocytes from PXB mouse (liver), hiPS-derived intestinal cell (intestine)	2D	100 μL/min flow rate	[73]
				Suggesting that the liver function is enhanced through gut–liver interactions	
Lung	syringe pump	L02 (liver), A549 and HFL-1 (lung)	3D	Various unidirectional flow rates of 1, 10, 20, 30, and 40 μL/min	[74]
				Analyzing the mechanisms of lung cancer metastasis to the liver and evaluating the cancer treatment efficacy of tirapazamine	
Kidney	peristaltic flow	primary human cell (liver and kidney)	3D	Pump operation at 0.5 Hz at 500 mbar pressure and 500 mbar vacuum	[75]
				Evaluating the renal therapeutic effect and distribution of mesenchymal stromal cell-derived small extracellular vesicles (EVs) to liver compartments	
Skin	peristaltic flow	HPR116, HHSteC (liver), EpiDerm™ (skin)	3D	Pump operation at 0.5 Hz at 350 mbar pressure and 300 mbar vacuum	[76]
				Metabolomic analysis of the hair dye 4-amino-2-hydroxytoluene	

## Data Availability

Not applicable.
